# The Silent Burden: Investigating Post-Traumatic Stress Disorder and Social Isolation Among Healthcare Workers During COVID-19

**DOI:** 10.3390/healthcare12232360

**Published:** 2024-11-25

**Authors:** Mariusz Goniewicz, Anna Włoszczak-Szubzda, Ahmed M. Al-Wathinani, Krzysztof Goniewicz

**Affiliations:** 1 Department of Emergency Medicine, Medical University of Lublin, 20-081 Lublin, Poland; mariusz.goniewicz@umlub.pl; 2 Faculty of Human Sciences, WSEI Academy, 20-209 Lublin, Poland; anna.wloszczak-szubzda@wsei.lublin.pl; 3 Department of Emergency Medical Services, Prince Sultan bin Abdulaziz College for Emergency Medical Services, King Saud University, Riyadh 11451, Saudi Arabia; 4 Department of Security, Polish Air Force University, 08-521 Deblin, Poland

**Keywords:** COVID-19, healthcare personnel, PTSD, occupational stress, social stigma, mental health, healthcare disparities, psychological resilience

## Abstract

**Background**: The COVID-19 pandemic has significantly impacted the mental health of healthcare professionals, particularly nurses and paramedics. This study investigates the prevalence of Post-Traumatic Stress Disorder (PTSD) and the impact of social ostracism on psychological distress among healthcare workers (HCWs) in Poland, with a focus on exploring the interplay between professional and social factors contributing to their stress. **Methods**: A cross-sectional survey was conducted between March 2021 and February 2022 with 852 HCWs from four Polish provinces. PTSD symptoms were measured using the PTSD Checklist—Civilian Version (PCL-C), and social ostracism was assessed through a custom-designed questionnaire. **Results**: Of the participants, 14.1% reported experiencing social ostracism, and 4.9% observed such experiences among colleagues. Those who experienced or witnessed ostracism reported significantly higher PTSD symptoms (*p* < 0.001). Concerns about personal health and the well-being of older individuals were strongly associated with increased PTSD severity, while concerns for household members were not. **Conclusions**: Social ostracism exacerbates the psychological burden on healthcare workers, contributing to higher levels of PTSD. This study highlights the need for targeted mental health interventions and support systems, including resilience training and stigma reduction initiatives, to address these challenges. Future research should explore cross-national comparisons and long-term psychological effects among diverse healthcare populations.

## 1. Introduction

The COVID-19 pandemic drastically impacted healthcare systems globally, placing unprecedented psychological burdens on healthcare workers (ss), particularly those on the front lines [1,2]. Post-Traumatic Stress Disorder (PTSD) emerged as a significant mental health risk due to continuous trauma exposure [3,4]. In line with DSM-5-TR criteria, PTSD is characterized by symptoms in four main clusters: intrusion, avoidance, negative mood alterations, and heightened arousal [5]. Previous crises, such as those of SARS and H1N1, also highlighted PTSD among HCWs, but COVID-19’s prolonged duration and unique social stressors amplified these risks [6,7].

In addition to PTSD, healthcare workers faced heightened levels of depression and anxiety due to sustained high-stress environments and prolonged social isolation [2]. These additional psychological challenges compounded the mental health burden among frontline workers, highlighting the need for a comprehensive approach to understanding their well-being.

While previous health crises also impacted healthcare workers’ mental health, the COVID-19 pandemic introduced several unprecedented factors. Its prolonged duration, coupled with recurring waves of infections, created a sustained high-stress environment that few healthcare workers had previously encountered [1,2]. Additionally, COVID-19’s impact on social interactions—through widespread lockdowns, mandatory social distancing, and changes in workplace dynamics—exacerbated feelings of isolation and social strain among frontline workers [4]. These unique challenges intensified the psychological toll on healthcare professionals, who not only faced physical risks but also endured ongoing social and emotional pressures. Addressing these compounded factors is critical, not only for the mental well-being of healthcare workers but also for maintaining the efficiency of health systems during crises

In addition to the risks associated with PTSD, healthcare workers also reported increased levels of depression and anxiety throughout the COVID-19 pandemic [3]. The unrelenting demands of high-pressure environments, combined with the isolation imposed by safety measures, contributed to a diverse spectrum of psychological challenges. These factors further amplified the mental health burden on frontline workers, underscoring the urgency of addressing their well-being through multifaceted and comprehensive interventions.

The COVID-19 pandemic also introduced unique social stressors, notably social ostracism, characterized by public and personal distancing from HCWs due to contagion fears [8]. This phenomenon, marked by exclusion from colleagues and communities, compounded feelings of vulnerability and intensified psychological distress. Social ostracism’s impact on mental health remains underexplored, despite its potential to exacerbate PTSD symptoms.

Another social factor was the divide in public perception caused by COVID-19 vaccines. In Poland, some healthcare workers faced scrutiny and stigma due to vaccine hesitancy, which, coupled with ethical concerns around vaccine mandates and public resistance, influenced their mental health and social standing [9]. This dual perception—of healthcare workers as both protectors and skeptics—likely contributed to additional stressors, especially for those working directly with COVID-19 patients [10,11,12].

This study is set within the Polish healthcare system, which faced considerable strain during the COVID-19 pandemic. Polish healthcare workers expressed not only concerns about their health and that of their families but also a growing sense of stigma linked to their profession during this period [13]. These concerns offer a unique context for understanding the compounded stressors faced by healthcare professionals in Poland and highlight the need for targeted support mechanisms that address both mental health and social factors during health crises.

In Poland, cultural factors play a significant role in shaping perceptions of healthcare professionals and mental health [14]. Healthcare workers are often regarded as essential yet vulnerable figures, especially during crises like the COVID-19 pandemic, where their close contact with patients heightened public fears of contagion [15,16,17,18]. Additionally, societal stigma surrounding mental health issues in Poland can deter individuals from seeking psychological support, exacerbating feelings of isolation and stress. These factors contribute to a complex social environment that influences both the mental well-being of healthcare workers and their public reception, underscoring the need for culturally sensitive mental health interventions.

While prior research has extensively documented the psychological toll of the COVID-19 pandemic on healthcare workers, our study uniquely examines the impact of social ostracism—a phenomenon exacerbated by fears of contagion—on mental health outcomes, particularly PTSD symptoms. This study’s focus on healthcare workers in Poland provides a distinct perspective on how social and cultural factors, including stigma related to vaccine hesitancy and professional exposure, contribute to the psychological distress experienced by frontline workers. By analyzing these factors, our research offers novel insights into an underexplored dimension of healthcare worker mental health during the pandemic, which may inform tailored interventions for this population.

This study aims to assess the prevalence of PTSD and to examine the impact of social ostracism on psychological distress among HCWs in Poland. Specifically, it seeks to quantify PTSD symptom prevalence, investigate the relationship between social ostracism and these symptoms, and explore how both professional and social factors contribute to the psychological stress experienced by healthcare workers. Additionally, the study will provide recommendations for mental health interventions and support systems to effectively address these challenges.

## 2. Materials and Methods

### 2.1. Location of the Study

This study was conducted between March 2021 and February 2022, targeting HCWs engaged in direct patient care across Poland. The survey was distributed to medical facilities in four distinct provinces: Mazovia, Łódź, Świętokrzyskie, and Lublin. These regions were chosen to reflect diverse healthcare settings within the country, capturing the range of experiences faced by HCWs during the COVID-19 pandemic. Due to pandemic safety measures, the research was conducted primarily through online platforms, ensuring widespread participation while adhering to health protocols.

### 2.2. Diagnostic Tools and Questionnaire

#### 2.2.1. PTSD Checklist—Civilian Version (PCL-C)

The PCL-C, a standardized self-report measure designed to assess PTSD symptoms based on DSM-IV criteria, was adapted for use in this study. Although the DSM-5-TR is now the standard for diagnosing PTSD, the PCL-C remains widely validated and used across various populations due to its reliability. We acknowledge that the DSM-5-TR includes four symptom clusters instead of three, which the PCL-C does not fully align with. However, given the global acceptance and proven reliability of the PCL-C, it was deemed appropriate for our study context. Future studies may consider DSM-5-TR-aligned tools to facilitate deeper comparisons.

The PCL-C includes 17 items rated from 1 (not at all) to 5 (extremely), with total scores ranging from 17 to 85. Higher scores indicate greater PTSD symptom severity. The scale assesses three key domains: re-experiencing, avoidance, and hyperarousal. While the tool has not been formally validated in the Polish population, its extensive global use, along with pilot testing in this study, supports its application. The internal consistency of the PCL-C in this study, as measured by Cronbach’s alpha, was 0.86, indicating satisfactory reliability.

#### 2.2.2. Custom-Designed Questionnaire

In addition to the PCL-C, a custom-designed questionnaire was developed based on in-depth interviews with healthcare personnel to capture the unique concerns of HCWs during the COVID-19 pandemic. The questionnaire included both closed-ended and Likert scale questions, which focused on four key areas: experiencing social ostracism, fears for personal health and life, concerns for the health and lives of family members, and concerns for the well-being of older acquaintances.

Closed-ended questions were designed to provide straightforward responses from participants. For instance, participants were asked, “Did you encounter any form of social ostracism due to your work during the pandemic?” with response options of “Yes”, “No”, or “Not me, but my colleagues did”. To further assess the intensity of participants’ concerns, Likert-scale-type questions were utilized. Participants were asked to rate their level of concern on a 5-point scale, ranging from 1 (not concerned at all) to 5 (extremely concerned). For example, one of the Likert-scale-type questions was, “How concerned are you about your personal health and life during the COVID-19 pandemic?” Additional Likert items included concerns such as “How concerned are you about the health and life of your household members?” and “To what extent do you worry about the well-being of older individuals in your environment?”.

The custom-designed questionnaire underwent pilot testing with a sample of 50 healthcare workers to ensure clarity, cultural relevance, and appropriateness for the target population. The feedback from pilot participants was used to refine the wording of specific questions, ensuring that they were understandable and applicable to the Polish healthcare context. In addition, expert judges reviewed the content to evaluate its validity and relevance to the study’s objectives. Their input was instrumental in ensuring that the questionnaire adequately captured the key concerns of healthcare workers during the pandemic.

While the custom questionnaire was not formally validated in the Polish population, the pilot testing and expert review provided an initial measure of its reliability. The Cronbach’s alpha value for internal consistency of the custom questionnaire was calculated at 0.82, indicating satisfactory reliability. However, we acknowledge that the absence of a formal validation process remains a limitation. Future studies should consider conducting comprehensive validation procedures to improve the cultural and linguistic appropriateness of the questionnaire across diverse healthcare settings.

#### 2.2.3. Presentation of Variables

The study aimed to capture a broad range of concerns and experiences related to the psychological well-being of healthcare workers during the COVID-19 pandemic. To achieve this, we employed both categorical and Likert-scale-type responses in the custom-designed questionnaire. The rationale behind this mixed approach was to accommodate the different natures of the variables being measured.

Categorical responses were used for questions that required straightforward, factual information. For example, social ostracism, a key variable in the study, was assessed using a closed-ended question: “Did you encounter any form of social ostracism due to your work during the pandemic?” Participants responded with one of three options: Yes, No, or Not me, but my colleagues did. These categorical responses provided a clear indication of whether participants or their colleagues had directly experienced social ostracism, allowing us to quantify its occurrence across the sample.

On the other hand, Likert-scale-type responses were used to assess the intensity of subjective experiences, such as concerns related to health and well-being. For example, participants were asked to rate their level of concern about their personal health during the pandemic on a 5-point scale, ranging from 1 (not concerned at all) to 5 (extremely concerned). This approach allowed us to capture the degree of concern participants felt, offering a more nuanced view of their psychological state.

The use of both categorical and Likert-scale-type responses was intentional, as it enabled us to quantify both the presence of specific experiences (e.g., social ostracism) and the intensity of subjective concerns. In the analysis, categorical variables were treated as distinct groups for comparison, while Likert-scale-type responses were used to evaluate the degree of psychological impact within those groups. For example, participants who reported experiencing social ostracism were compared to those who did not, with PTSD symptom intensity measured using the Likert scales. This mixed-method approach provided a comprehensive understanding of both the occurrence of social ostracism and its associated psychological impact.

#### 2.2.4. Reliability and Pilot Testing

Both instruments—the PCL-C and the custom-designed questionnaire—were subjected to pilot testing with a sample of 50 healthcare workers to assess internal consistency and relevance. The Cronbach’s alpha values were calculated for both tools, yielding 0.86 for the PCL-C and 0.82 for the custom-designed questionnaire, indicating satisfactory internal consistency for both instruments. Feedback from the pilot testing led to minor adjustments in the wording of certain questions to ensure clarity and applicability within the Polish healthcare context.

### 2.3. Study Population and Sampling

The initial participant pool consisted of 1022 healthcare workers across four provinces in Poland. Participants were included based on the criteria of being directly involved in patient care during the COVID-19 pandemic. The inclusion criteria required that participants must have been employed in healthcare facilities within the study regions during the period from March 2021 to February 2022.

Of the initial 1022 healthcare workers who participated in the study, 170 participants were excluded due to incomplete survey responses. Incomplete responses were defined by several factors: participants who failed to answer more than 20% of the survey questions, particularly key sections such as those assessing PTSD symptoms using the PCL-C or providing essential demographic data (e.g., age, gender, professional role). Surveys were also excluded if critical information related to the healthcare setting or experiences of social ostracism was missing or incomplete. Additionally, responses that contained inconsistencies, such as contradictory answers or extreme values indicating low engagement or potential misunderstandings during the survey process, were also excluded. These exclusions ensured the integrity and robustness of the data used for analysis. After applying these criteria, 852 participants were retained for the final analysis.

### 2.4. Statistical Analysis

Descriptive statistics were calculated for demographic data, and inferential analyses were conducted to explore relationships between variables. Pearson’s r was used for correlation analyses. Given the nature of the data and the potential for non-normal distributions, non-parametric tests such as the Mann–Whitney U test and Kruskal–Wallis test were employed to compare groups based on experiences of social ostracism and PTSD symptom severity.

Post hoc comparisons were performed using Bonferroni correction to control for Type I error. The statistical significance threshold was set at α = 0.05. IBM SPSS Statistics, version 26, was used for all analyses. The rationale for using non-parametric tests stems from the ordinal nature of some variables (e.g., Likert scale responses), and the unequal group sizes between those reporting social ostracism and those who did not.

### 2.5. Ethical Considerations

The study was conducted in compliance with the ethical principles stipulated by Polish law. According to the Act of 5 December 1996 on the professions of doctors and dentists (published on the Polish Sejm site: https://isap.sejm.gov.pl/isap.nsf/DocDetails.xsp?id=WDU19970280152, accessed on 21 June 2024), research of this nature does not fall under the definition of a medical experiment. Therefore, it is exempt from the requirement of obtaining ethics approval, and consequently, formal opinion from the Institutional Review Board (IRB) at the Medical University of Lublin or the Bioethics Committee was not necessary.

Despite this exemption, all participants were thoroughly informed about the study, its objectives, and the voluntary nature of their participation. We ensured strict confidentiality and secure data storage for all collected data. Participants provided informed consent, acknowledging their right to withdraw from the study at any time without any consequences. This approach aligns with the ethical guidelines of the Declaration of Helsinki, ensuring respect for individual autonomy and the ethical conduct of the study.

### 2.6. Limitations of the Methodology

The use of online surveys, while necessary due to COVID-19 restrictions, presents potential limitations, such as non-response bias and accessibility issues. Some healthcare workers, especially those in remote areas, may have had limited internet access, which could affect the generalizability of the findings. Additionally, self-reported data are subject to biases such as recall bias and social desirability bias, which may impact the accuracy of the responses.

## 3. Results

### 3.1. Demographic Overview

Our study included 852 healthcare workers, primarily women (88.1%) and nurses (86.6%). The average age was 39.02 years (±10.02), with work experience ranging from less than a year to 41 years (average: 12.90 years ± 11.44). This over-representation of female nurses aligns with previous research that shows that female healthcare professionals tend to report higher levels of psychological stress and PTSD, especially in high-stress settings like public health crises.

Additionally, 91.8% of the participants reported active engagement in healthcare duties during the COVID-19 pandemic. Notably, 21.9% worked directly in COVID-19 wards, while 40.3% were in non-COVID-19 wards. Emergency department workers accounted for 8.2% of the sample, highlighting their involvement in critical, high-risk environments.

The participant demographics are summarized in Table 1, which outlines the gender distribution, age range, professional roles, and work settings. These data help contextualize the findings, particularly the heightened psychological stress observed among certain groups, such as frontline workers and those in COVID-19 wards.

### 3.2. Experiences of Social Ostracism

The majority of participants (80.8%) did not report experiencing social ostracism due to their work during the pandemic. However, 14.1% of healthcare workers reported personal experiences of ostracism, and 4.9% observed such exclusion among their colleagues. While the number of participants directly affected by social ostracism was relatively small, its impact is significant.

Our analysis demonstrated a clear link between social ostracism and heightened PTSD symptoms. Participants who experienced or observed ostracism reported higher levels of psychological distress, particularly in the areas of intrusion, avoidance, and heightened arousal. This suggests that even a smaller proportion of workers facing exclusion can have a disproportionate psychological burden, highlighting the need for further research into how workplace culture and societal stigma exacerbate mental health issues during public health emergencies.

**Figure 1:**
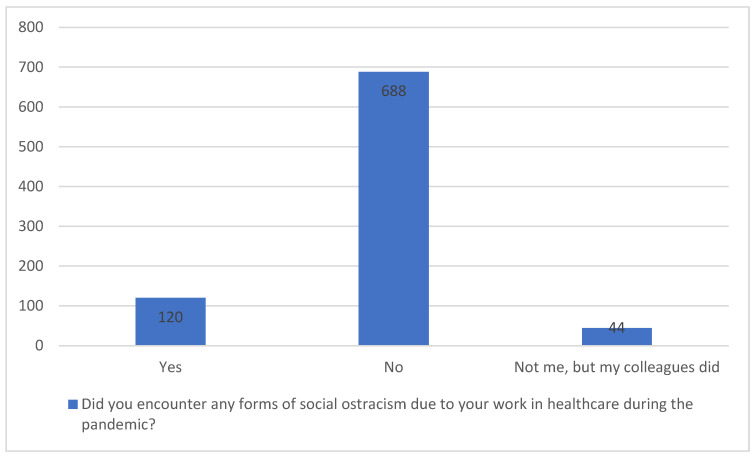
Incidence of social ostracism experienced by healthcare workers during the COVID-19 pandemic. Legend: “Yes” represents healthcare workers who personally experienced social ostracism due to their role during the pandemic. “No” indicates healthcare workers who did not encounter any form of social ostracism. “Not me, but my colleagues did” refers to respondents who did not personally experience ostracism but reported that their colleagues were subject to it.

These findings point to an urgent need for targeted interventions to address the psychological and social challenges healthcare workers face, particularly those who experience stigma and exclusion (see Figure 1 for a visual representation of ostracism experiences). This figure illustrates survey responses regarding the experience of social ostracism among healthcare workers during the COVID-19 pandemic. The majority (688) reported no personal experience of ostracism, while a subset (120) did experience it. Additionally, 40 respondents indicated that their colleagues, rather than themselves, faced social exclusion. These findings highlight the significant social pressures placed on healthcare professionals due to their frontline roles in managing the pandemic.

### 3.3. Analysis of PTSD Symptoms and Social Ostracism

In this section, we examined the primary concerns of all participants, including those who reported experiencing social ostracism and those who did not. Our goal was to capture a holistic view of the worries and fears that affected healthcare workers during the pandemic. This analysis helps contextualize the broader psychological stressors, beyond just the experience of social ostracism.

The most frequently reported concern was for the health and lives of older individuals, expressed by 47.5% (N = 405) of participants. Nearly as many participants (46.8%, N = 399) were worried about their own personal health and life. Additionally, 36.6% (N = 312) voiced concerns about the health and lives of household members. Interestingly, 15.1% (N = 129) reported no specific concerns, while 1.4% (N = 12) mentioned other worries not explicitly listed in the questionnaire.

It is important to note that the percentages are based on a total of 1257 responses, as participants could select multiple concerns. Therefore, these percentages represent the distribution of concerns across all responses rather than the proportion of the total sample.

**Figure 2:**
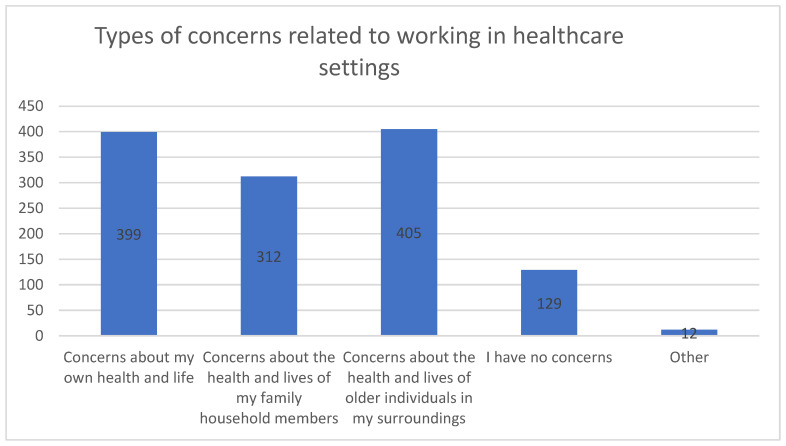
Types of concerns reported by healthcare workers related to COVID-19 exposure. Legend: “Concerns about my own health and life” represents healthcare workers who reported anxiety about their personal health and safety. “Concerns about the health and lives of my household members” includes those worried about the well-being of people they live with. “Concerns about the health and lives of older individuals in my surroundings” indicates concern for elderly individuals whom healthcare workers interact with or care for. “I have no concerns” represents those who reported feeling unaffected by health-related anxieties in the context of their work. “Other” includes any additional concerns not categorized here.

These findings highlight the pervasive nature of healthcare workers’ fears during the pandemic, emphasizing that concerns about both personal health and the well-being of others were top-of-mind for many. The intersection of these worries, combined with the experience of social ostracism, contributes to heightened PTSD symptoms. Figure 2 provides a visual representation of the different types of concerns reported by participants. This figure displays the types of health-related concerns reported by healthcare workers during the COVID-19 pandemic. The most common concerns were related to the health and safety of older individuals in their surroundings (405) and their own health and life (399). Many also expressed worries about the well-being of household members (312). A smaller group (129) reported no specific concerns, while a few (22) indicated other types of concerns. These results highlight the breadth of anxieties experienced by healthcare workers, reflecting the multifaceted impact of the pandemic on those in healthcare settings.

### 3.4. PTSD Symptoms and Departmental Differences

In our evaluation of PTSD symptoms using PCL-C scores, a substantial portion of participants exhibited symptoms indicative of significant psychological distress during the pandemic. Notably, healthcare staff working in COVID-19 wards exhibited significantly higher levels of PTSD symptoms compared to their counterparts in non-COVID-19 departments. This finding underscores the increased psychological burden on staff directly involved in treating and caring for COVID-19 patients. The statistically significant differences (*p* < 0.05) observed in PTSD symptom severity between these groups highlight the unique stressors faced by healthcare workers in high-risk settings. 

In addition to elevated PTSD symptoms, participants from COVID-19 wards also reported more frequent experiences of social ostracism and heightened work-related anxiety, further amplifying their psychological stress. These differences point to the need for tailored mental health interventions for healthcare workers in high-stress departments, especially those directly handling COVID-19 cases. Targeted support systems are essential to address both the workplace-specific challenges and the social exclusion these workers may face.

### 3.5. Differences in PTSD Symptoms Based on Social Ostracism Experiences

In our analysis of PTSD symptoms relative to experiences of social ostracism, we sought to determine whether healthcare workers who either personally experienced or witnessed ostracism exhibited differing levels of PTSD symptom intensity compared to those who did not encounter ostracism. Using the Kruskal–Wallis test, a non-parametric method for comparing multiple groups, we found statistically significant differences (*p* < 0.05) in PTSD symptoms across all measured indicators, including intrusion, avoidance, and hyperarousal.

Healthcare workers who reported experiencing social ostracism, either directly or in their environment, consistently showed higher levels of PTSD symptoms. These elevated levels were observed particularly in the domains of intrusion (re-experiencing traumatic events) and avoidance (efforts to avoid reminders of the trauma), which suggests that social exclusion can compound the psychological impact of working in high-stress healthcare environments.

Interestingly, the analysis revealed no significant differences in the severity of PTSD symptoms between those who personally experienced ostracism and those who only witnessed their colleagues being ostracized. Both direct and indirect exposure to social ostracism during the pandemic were associated with negative effects on mental health. The data indicates significant correlations between workplace stigma, social exclusion, and PTSD symptom severity (Table 2).

### 3.6. PTSD Symptoms in Relation to Specific Concerns

This section examines the relationship between PTSD symptoms and the specific concerns healthcare workers expressed related to their healthcare roles during the COVID-19 pandemic. Participants were queried about their fears regarding personal health, the health of household members, and the well-being of older individuals in their immediate environment.

Our analysis revealed that healthcare workers who reported concerns about their own health or the health of older individuals exhibited significantly higher PTSD symptoms across all PTSD indicators (intrusion, avoidance, heightened arousal). These individuals showed higher levels of stress and psychological distress in comparison to those who did not express these specific concerns. Interestingly, concerns about the health and life of household members did not significantly influence the severity of PTSD symptoms, suggesting that worries about oneself and older individuals are more strongly associated with increased psychological burden.

The results of this analysis are detailed in Table 3 below, which presents the relationship between PTSD symptom severity (intrusion, avoidance, and heightened arousal) and specific health-related concerns among healthcare workers. The table compares mean PTSD symptom scores (M) and standard deviations (SD) for individuals with and without particular health concerns, such as concerns about their own health or the health of older individuals. Statistical values, including *t*-values, *p*-values, 95% confidence intervals, and effect sizes (Cohen’s d), are provided to indicate the strength and significance of these relationships. For example, healthcare workers with concerns about their own health reported significantly higher intrusion and avoidance symptoms compared to those without these concerns (*p* < 0.001). This analysis highlights how specific health-related worries correlate with variations in PTSD symptom severity.

The concerns related to personal health and the health of older individuals in the environment significantly contributed to the increased intensity of PTSD symptoms. These findings suggest that healthcare workers with these specific anxieties are experiencing a higher psychological burden, which is critical for understanding how to tailor mental health interventions for healthcare workers. Targeted psychological support aimed at addressing these particular fears could help mitigate the intensity of PTSD symptoms and improve overall mental well-being in healthcare professionals.

This analysis emphasizes the importance of addressing specific stressors in healthcare workers’ lives, particularly during times of public health emergencies. By recognizing and focusing on the most impactful concerns, mental health interventions can be more effectively designed to alleviate the psychological toll on frontline workers.

## 4. Discussion

Our findings contribute to the growing body of literature on the mental health challenges faced by healthcare workers during pandemics, specifically addressing the unique impact of social ostracism. Unlike previous studies that primarily focus on burnout and generalized stress, this research highlights social exclusion as a specific, culturally influenced factor that compounds PTSD symptoms among healthcare workers [19]. The Polish context, where some workers faced dual perceptions as both essential workers and potential contagion risks, underscores the need for culturally and socially sensitive mental health support during health crises [20].

This study investigated the psychological impact of the COVID-19 pandemic on healthcare workers, with a focus on the correlation between social ostracism and PTSD symptoms. Our findings revealed that 14.1% of healthcare workers experienced social ostracism due to their profession, with an additional 4.9% reporting that their colleagues encountered such exclusion. This highlights the significant social pressures healthcare workers faced during the pandemic, which contributed to elevated PTSD symptoms, particularly in the domains of intrusion, avoidance, and heightened arousal [21,22,23,24,25]. These results emphasize the compounded psychological stress within healthcare work environments during the global health crisis.

Social ostracism, as a key factor, exacerbated the mental health challenges of healthcare workers, which aligns with global research on the heightened risks frontline workers face during pandemics [26,27]. However, our study uniquely highlights social ostracism as an underexplored element contributing to the worsening of PTSD symptoms. The stigmatization of healthcare workers due to fears of contagion adds a layer of psychological vulnerability, underscoring the need for mental health interventions that address both direct stressors and social exclusion.

The stigma and public perception of healthcare workers as potential sources of contagion had far-reaching consequences. Stigmatization often discouraged healthcare workers from seeking help, as fears of judgment or further isolation inhibited their willingness to access mental health resources [28]. This reluctance to seek support likely intensified their feelings of isolation and contributed to a cumulative psychological burden, adding to the stress of working in a high-risk environment. These outcomes underscore the critical need for mental health interventions that not only address direct stressors but also actively combat stigma, fostering an environment where healthcare workers feel supported and safe to access necessary psychological assistance.

The relationship between social ostracism and PTSD can be further understood through theoretical frameworks such as social identity theory and stigma theory. According to social identity theory, healthcare workers derive part of their self-concept from their professional roles. However, during the pandemic, the public’s fear of contagion led to healthcare workers being perceived as potential carriers of the virus, disrupting their social identity and contributing to feelings of exclusion. Stigma theory explains how the fear and misunderstanding of the virus labeled healthcare workers as dangerous, resulting in their social ostracism [29]. These factors compounded the psychological stress experienced by healthcare workers and contributed to the onset or worsening of PTSD symptoms. Understanding these dynamics highlights the need for mental health interventions that address both the professional challenges, and the societal stigma faced by healthcare workers during health crises.

When contextualizing these findings within global research, the prevalence of social ostracism observed in our study is notably higher than in previous pandemics, such as those of SARS and MERS, where lower rates of social exclusion were reported among healthcare workers [30]. This difference may reflect specific cultural or societal factors in Poland that heightened the social stigma against healthcare workers during the COVID-19 pandemic. Such stigmatization amplifies the psychological toll on healthcare workers, reinforcing the need for public health education to reduce stigma during health emergencies.

The severity of PTSD symptoms observed in this study is consistent with findings from other regions, such as Greece and Italy, where healthcare workers experienced similar levels of psychological distress during the COVID-19 pandemic [31,32,33]. However, this study’s focus on social ostracism adds a critical social dimension to understanding the mental health burden faced by healthcare workers. Addressing this dynamic is essential for developing comprehensive mental health interventions.

The relationship between social ostracism and PTSD can be understood through frameworks of social identity and stigma, which suggest that public fears of contagion likely fueled the exclusion of healthcare workers. This social exclusion compounds stress and contributes to the development of PTSD, particularly in high-stress professions like those in healthcare [34,35].

An additional factor affecting the mental health of healthcare workers during the COVID-19 pandemic was the escalation of patient tension, which correlated with instances of aggression directed at healthcare professionals. The uncertainty, fear, and frustration experienced by patients during the pandemic often manifested as hostility, placing HCWs in challenging and, at times, confrontational situations. This increase in patient aggression added another layer of psychological strain, further exacerbating PTSD symptoms and feelings of vulnerability among HCWs. 

Implementing systematic incident reporting and analysis, as highlighted in the study by Male et al., is crucial for identifying risk factors and developing targeted interventions to mitigate patient aggression [36]. Such preventive systems can enhance the safety and well-being of HCWs, ensuring they are better equipped to handle high-stress interactions.

To mitigate these psychological impacts, targeted interventions are crucial. Strategies such as resilience training, mindfulness practices, and enhanced workplace support systems can alleviate the burden on healthcare workers [37,38,39]. Public health initiatives aimed at reducing stigma and promoting understanding for healthcare workers are equally important. Institutional support, as emphasized by Buselli et al., plays a pivotal role in addressing the mental health needs of healthcare workers during pandemics [40].

The broader social implications of this study highlight a disconnect between public perceptions of healthcare workers and the realities they face. Educational campaigns and media engagement are needed to reshape public attitudes and foster a culture of respect and support for healthcare workers during health crises [41,42,43]. Fostering resilience through interventions like mindfulness and resilience training has proven effective in reducing stress and improving mental health outcomes [44]. Addressing social ostracism requires both public health campaigns to reduce stigma and workplace policies that protect healthcare workers from exclusion [45,46].

Future research should explore the long-term psychological effects of the COVID-19 pandemic on healthcare workers. Longitudinal studies could provide valuable insights into the persistence of PTSD and other mental health challenges over time. Additionally, cross-country comparative studies could shed light on how different healthcare infrastructures and cultural factors contribute to social ostracism and mental health outcomes, informing best practices for mental health support [47].

The findings of this study have significant implications for healthcare policy and workforce management. Comprehensive mental health policies integrated into healthcare systems, including regular mental health assessments and strategic workforce management, are necessary to reduce the risk of burnout and PTSD among healthcare workers. These measures would also improve patient care by ensuring that healthcare professionals remain mentally healthy and resilient [48,49,50,51].

In conclusion, this study highlights the complex interplay between social ostracism and PTSD among healthcare workers during the COVID-19 pandemic. As healthcare systems prepare for future health crises, the lessons from this pandemic should guide the development of more robust mental health support systems for healthcare professionals, ensuring their well-being in times of crisis.

## 5. Limitations

This study has several limitations that should be considered. First, the participant demographics predominantly included female nurses, limiting the generalizability of the findings to other healthcare professionals such as physicians or paramedics. A broader representation of healthcare roles and a more balanced gender distribution would strengthen future research.

Second, the study was conducted solely in Poland, making it culturally and regionally specific. While providing important insights into the Polish healthcare context, these findings may not be fully applicable to other countries with different healthcare infrastructures and social attitudes. Cross-national comparisons in future studies could offer more generalizable insights.

Third, the use of online data collection methods may have introduced selection bias, potentially excluding participants who were less familiar with digital platforms. This could affect the representativeness of the sample, particularly among older or rural healthcare workers. A combination of data collection methods in future research could mitigate this limitation.

Another limitation is the reliance on self-reported data, which is susceptible to recall bias and social desirability bias. Additionally, the cross-sectional design of the study prevents causal inferences regarding the long-term mental health impacts of the pandemic. Future longitudinal studies would better capture the evolution of psychological symptoms over time.

Lastly, the use of the PTSD Checklist—Civilian Version (PCL-C) focused primarily on PTSD symptoms, potentially overlooking other mental health issues like anxiety or depression. A more comprehensive mental health assessment would provide a fuller understanding of the psychological toll on healthcare workers.

These limitations suggest the need for future studies with a more diverse sample, broader mental health measures, and longitudinal designs to better capture the lasting impact of pandemics on healthcare workers.

## 6. Conclusions

This study highlights the significant psychological burden healthcare workers faced during the COVID-19 pandemic, particularly due to social ostracism and its strong correlation with heightened PTSD symptoms. With 14.1% of participants reporting experiences of social ostracism, this study emphasizes the compounded stress healthcare workers endured, both from the physical demands of their jobs and the emotional toll of social exclusion.

Our findings stress the need for comprehensive mental health support systems that go beyond direct pandemic-related stressors. Counseling services, stress management programs, and resilience-building interventions like mindfulness training are critical for enhancing long-term resilience. Addressing social ostracism should also involve public health campaigns aimed at reducing stigma and fostering societal support, alongside workplace policies that promote inclusion and safeguard healthcare professionals from isolation.

This study adds to the global understanding of the psychological impact of the pandemic on healthcare workers, particularly by providing insights from the Polish context. These findings should drive healthcare systems to prioritize the mental well-being of their staff, preparing them to face future global health challenges with the necessary support and resilience.

## Figures and Tables

**Table 1:** Participant demographic overview.

Demographic Feature	Count (N)	Percentage (%)
Gender		
− Women	751	88.1
− Men	101	11.9
Age Range		
− Avarage Age ± SD		39.02 ± 10.02 years
− Range	20–59 years	
Work Experience		
− Work Experience ± SD		12.90 ± 11.44 years
− Range	0.08–41 years	
Profession role		
− Nurse	704	86.6
− Paramedic	75	8.9
− Physician	8	0.9
− Medical Caregiver	14	1.6
− Other	51	6.0
Work Setting		
− Primary Healthcare	119	14
− Specialist Ambulatory Care	34	4.0
− Emergency Department	70	8.2
− Admissions Room	47	5.5
− Care and Treatment Institution	39	4.6
− Social Welfare Home	36	4.2
− Hospital (non-COVID-19 ward)	343	40.3
− Hospital (COVID-19 ward)	187	21.9
− Ambulance (non-COVID cases)	36	4.2
− Ambulance (COVID cases)	48	5.6
− Other	123	14.4
Worked During Pandemic?		
− Yes	782	91.8
− No	70	8.2
Total Participants	852	100

**Table 2:** Comparison of PTSD symptom severity among healthcare workers who experienced or witnessed social ostracism during the COVID-19 pandemic.

Did You Encounter Any Forms of Social Ostracism Due to Your Work in Healthcare During the Pandemic?	Yes (n = 120)	No (n = 688)	My Colleagues, Not Me (n = 42)	H(2)	*p*	η^2^
Intrusion	493.43 (Mdn = 11.00, IQR = 6.00)	407.32 (Mdn = 9.00, IQR = 4.00)	529.25 (Mdn = 10.00, IQR = 3.50)	20.69	<0.001	0.02
Avoidance	504.24 (Mdn = 16.00, IQR = 9.00)	405.33 (Mdn = 14.00, IQR = 7.75)	530.93 (Mdn = 17.00, IQR = 6.25)	24.82	<0.001	0.03
Heightened Arousal	518.30 (Mdn = 14.00, IQR = 7.00)	405.24 (Mdn = 11.00, IQR = 6.00)	492.30 (Mdn = 13.00, IQR = 8.00)	25.15	<0.001	0.03
Overall PTSD Score	515.72 (Mdn = 41.00, IQR = 21.00)	403.64 (Mdn = 34.00, IQR = 16.00)	525.86 (Mdn = 40.00, IQR = 18.50)	28.70	<0.001	0.03

Note: n = number of participants; Mdn = Median; IQR = Interquartile Range; H(2) = Kruskal–Wallis H statistic; *p* = Significance level; η^2^ = effect size (Eta Squared).

**Table 3:** PTSD symptom severity in relation to specific health-related concerns among healthcare workers.

Dependent Variable	Type of Concern	Absence of Concern (M, SD)	Presence of Concern (M, SD)	*t*-Value	*p*-Value	95% Confidence Interval	Cohen’s d
Intrusion	Concern about own health	9.31 ± 3.65	11.01 ± 4.00	6.47	<0.001	2.21 to 1.18	0.44
	Concern about older individuals	9.73 ± 3.74	10.52 ± 4.05	2.94	0.003	1.31 to 0.26	0.20
	No concerns	10.45 ± 3.90	8.19 ± 3.38	6.20	<0.001	1.55 to 2.98	0.59
Avoidance	Concern about own health	14.26 ± 5.50	15.93 ± 5.73	−4.34	<0.001	−2.43 to −0.92	0.30
	Concern about older individuals	14.59 ± 5.63	15.55 ± 5.68	2.47	0.014	1.72 to 0.20	0.17
	No concerns	15.42 ± 5.65	12.97 ± 5.35	4.57	<0.001	1.40 to 3.50	0.44
Heightened Arousal	Concern about own health	12.03 ± 4.56	13.53 ± 4.54	4.78	<0.001	2.11 to 0.88	0.33

## Data Availability

The datasets used and/or analyzed during the current study are available from the corresponding author on reasonable request.
